# Epistemologies of evidence-based medicine: a plea for corpus-based conceptual research in the medical humanities

**DOI:** 10.1007/s11019-021-10027-2

**Published:** 2021-05-31

**Authors:** Jan Buts, Mona Baker, Saturnino Luz, Eivind Engebretsen

**Affiliations:** 1 Boğaziçi University, Istanbul, Turkey; 2 University of Oslo, Oslo, Norway; 3 Usher Institute, Edinburgh Medical School, University of Edinburgh, Edinburgh, Scotland, UK; 4 Faculty of Medicine, University of Oslo, Blindern, BOX 1078, 0316 Oslo, Norway

**Keywords:** Evidence-based medicine, Corpora, Basic concept, Social epistemology, Evidence

## Abstract

Evidence-based medicine has been the subject of much controversy within and outside the field of medicine, with its detractors characterizing it as reductionist and authoritarian, and its proponents rejecting such characterization as a caricature of the actual practice. At the heart of this controversy is a complex linguistic and social process that cannot be illuminated by appealing to the semantics of the modifier *evidence-based*. The complexity lies in the nature of *evidence* as a basic concept that circulates in both expert and non-expert spheres of communication, supports different interpretations in different contexts, and is inherently open to contestation. We outline a new methodology that combines a social epistemological perspective with advanced methods of corpus linguistics and elements of conceptual history to investigate this and other basic concepts that underpin the practice and ethos of modern medicine. The potential of this methodology to offer new insights into controversies such as those surrounding EBM is demonstrated through a case study of the various meanings supported by *evidence* and *based*, as attested in a large electronic corpus of online material written by non-experts as well as a variety of experts in different fields, including medicine.

## Introduction

Ever since the rise of modern medical science in the early nineteenth century, medical practice has been closely associated with science and research, and *evidence* has grown to become a key term in modern medical discourse. This has been particularly true since the emergence of the evidence-based medicine (EBM) movement in the early 1990s. The basic idea in EBM is that clinical and health policy decisions should not be based merely on intuition, expert opinion, or pathophysiological reasoning. Such sources are considered potentially biased and unreliable. Evidence-based decisions should integrate expertise and patient preferences with the “best available external evidence from systematic research” (Sackett et al. 1996, 71: 78). Evidence, in the EBM sense of the word, is mainly associated with randomized controlled trials (RCTs), i.e. comparative experimental intervention studies, which are considered the ‘gold standard’ for assessing cause-effect relationships between an intervention and its outcome; the findings generated by RCTs are likely to be closer to the true effect than the findings generated by other research methods (Evans 2003). Ideally, such trials should be summarized through systematic reviews and form the basis for clinical guidelines for clinicians and decision makers following standardized procedures. Other research designs such as observational studies and even qualitative studies are officially included in the EMB framework, but in practice are treated as less reliable sources of evidence. Indeed, EBM has developed a framework for ranking evidence in a hierarchy that features simple observational methods at the bottom and moves on to increasingly rigorous methodologies, notably RCTs and systematic reviews, at the top.

This received ranking of evidence and the singular, restricted conceptualization of *evidence* it perpetuates are not always helpful, as the current crisis has amply illustrated. For instance, Greenhalgh (2020) has recently demonstrated that EBM fails to acknowledge the whole fabric of evidence relevant to the question of using facemasks as a measure against the spread of COVID-19. Although there are few relevant RCTs to warrant decisions on this issue, several other pertinent strands of evidence are available. Along with epidemiological studies, Greenhalgh (2020) refers to the physics of droplets and aerosols, the material science of masks, mathematical modelling, political and behavioural science, economics, and anthropology in order to answer various questions regarding the use of facemasks. In addition, she draws on semi-scientific sources such as handbooks, newspaper articles and patient experiences. These can all be considered sources of different types of evidence, she claims—although they address different purposes. An anthropological study about cultural attitudes or compliance does not measure the ‘true effects’ or statistical magnitude of an intervention, but that does not make its findings unreliable as evidence in every sense of the word. They provide evidence of a different kind.

The same argument may be extended to what constitutes evidence for non-academic groups, including groups that question and challenge conventional medicine. Anti-vaccine groups and various proponents of alternative medicine have contested the meanings and uses of *evidence* and associated concepts in EBM, partly in response to what they perceive as the hierarchical, authoritarian and impersonal character of the medical community (Browne 2018). These groups support their views through various practices of evidence, and many of them draw on knowledge sources associated with traditional academic institutions. EBM cannot understand the motivations of those promoting anti-vaccination theories if it simply dismisses their arguments as non-evidence. Without suggesting that the alternative basis of evidence these groups promote must be accepted, there is a need to identify and analyse the discursive means by which they support their claim to scientific legitimacy (Kata 2012). By understanding what concept of evidence informs their behaviour, the arguments put forward by these groups can be better addressed and questioned, and, where necessary, misinformation can be targeted by effective counter-information.

This article is a plea for initiating a new research strand within the medical humanities that combines advanced methods in corpus linguistics and critical discourse analysis with some of the assumptions of social epistemology and conceptual history in order to address two main aims. The first is to investigate the genealogy and contestation of a constellation of concepts that has underpinned the practice and ethos of modern medicine since the early nineteenth century. This requires access to a large body of electronic corpora of texts originating at different historical moments and drawn from a diverse range of genres, including medical journal articles, WHO and CDC reports, systematic reviews, guidelines and internet blogs. To demonstrate the potential of the proposed methodology in the absence of access to these resources, which we are planning to build and share with the research community in the near future, we will here present a case study exploring the variety and complexity of meanings currently associated with the concept of *evidence-based* in modern medicine, as well as the various forms of contestation it has invited within and beyond the medical community. We attempt to unpack this complex concept by analysing the patterning of its individual components (*evidence* and *based*), as attested in the Genealogies of Knowledge corpus of Internet English (see “Data, methods and theoretical underpinnings” section).

The second aim of our research group is to develop models for situated epistemologies and ontologies able to cope with the huge challenges to established frameworks of knowledge in modern medicine that the COVID-19 crisis has particularly thrown into relief in recent months. This is a more ambitious aim which we hope to pursue through a series of future case studies. For our immediate purposes, we focus mostly on demonstrating our proposed methodology and contributing to the (limited) literature on the meanings of *evidence-based* as a key concept in modern medicine and a growing range of other areas of practice (“Analysis” section).

## Data, methods and theoretical underpinnings

This article supports its plea for a new research strand in the medical humanities with a small case study of the linguistic patterning of *evidence-based* in a corpus of Internet English, and what this patterning reveals about the potential for this widely used term to generate different meanings in different contexts, in part because of the numerous meanings supported by each of its constituent elements (*evidence* and *based*). The analysis is rooted in the theoretical assumptions and draws primarily on the methodological innovations of two areas of scholarship: corpus linguistics (CL) and critical discourse analysis (CDA). Both insist on the primacy of attested uses of language, focus on identifying repeated patterns, and offer generalizations based on close analysis of such patterns. Corpus linguistics offers theoretical notions that assist in the analysis of the semantic and affective dimensions of key concepts such as *evidence* and *evidence-based,* as revealed in actual use across many texts, authors and historical periods, depending on the design of the corpora to which the analyst has access. These include, for example, the notion of collocation (the habitual co-occurrence of words), semantic preference (the tendency of a certain word to collocate with other words from a specific semantic set), and semantic prosody (collocational patterns that express the speaker or writer’s attitude or evaluation) (Sinclair 1991; Louw 2000).

Influenced by the work of Michel Foucault, CDA seeks to explain the conditions of possibility for the recurrent use of particular linguistic patterns by examining the social practices and ideological context in which they are embedded. CDA assumes that any discursive event is shaped by the institutions and social structures in which it is embedded but also shapes them; in other words, that “discourse is socially constitutive as well as socially conditioned” (Wodak and Fairclough 1997, p. 258). Corpus-based CDA integrates the two theoretical traditions to arrive at more holistic and detailed descriptions of a large body of data rather than limited samples of language in use, while maintaining the focus on discourse as constitutive of social relations. By combining large scale quantitative and qualitative analyses of language in use, Corpus-based CDA avoids the overemphasis on statistical methods typical of many corpus linguistic studies, including the very few that have recently been conducted in the field of medicine (Aiello and Simeone 2019). The emphasis on discourse as ‘structured forms of knowledge’ rather than text as a linguistic artefact means that Corpus-based CDA studies typically supplement statistical analyses with close examination of extended stretches of text, a methodology that is more consistent with the tenets of social epistemology and the objectives of the strand of research we advocate here.^1^

Work in Corpus-based CDA generally focuses on the ideological implications of linguistic patterns associated with lexical items such as *migrants* or *climate change* in the media (Baker et al. 2008), or metaphorical uses of language in specific sites of communication (Wei 2016). In the field of healthcare and education, Semino et al. (2015) explore the use of metaphors of Violence and Journey among cancer patients and health professionals on online forums, and Cleland and Palma (2018) examine the discourse of UK deans of medical schools, arguing that it leads to the othering of widening participation applicants and exacerbates social divides. Our proposed methodology goes beyond this type of analysis in one important respect.^2^ It focuses not on lexical items or metaphors but on basic concepts, as defined and understood in the field of conceptual history. Basic concepts have considerable currency in both specialist and public discourse. They “combine manifold experiences and expectations in such a way that they become indispensable to any formulation of the most urgent issues of a given time”, and “are always both controversial and contested” (Koselleck 1996, p. 64). Precisely because of these characteristics, they are “pivots around which all arguments turn”, and their history cannot be separated from the history of discourse, in Foucault’s sense of the term (ibid.:65). Given the entanglement of their scientific and public meanings and uses, a study of a basic concept such as *evidence* in medicine, for example, cannot be separated from its study in everyday, non-specialist use, nor can it be restricted to a specific genre or register. Our focus on basic concepts and constellations of concepts rather than lexical items or metaphors requires compiling thematic corpora that cut across many genres rather than, for instance, corpora solely constituted of newspaper articles or case reports.

Finally, the research approach suggested in this paper is theoretically situated within the broad and rather ambiguous tradition of social epistemology. This branch of epistemology was systematically developed by Steve Fuller in the late 1980s but was first conceptualized by the library scientist Jesse Shera, who defined it as “the study of knowledge in society” and suggested that its focus “should be upon the production, flow, integration, and consumption of all forms of communicated thought throughout the entire social fabric” (Shera 1970, p. 86). Social epistemology later took two divergent directions. According to one of its pioneers, Alan Goldman, social epistemology should maintain the truth-oriented ambition of traditional epistemology, but with an emphasis on collective agents, rather than individual agents: “social epistemology is, in the first instance, an enterprise concerned with how people can best pursue the truth […] with the help of, or in the face of, others” (Goldman and O’Connor 2019, p. 1). Fuller favours a different program that is more inspired by science and technology studies and a social constructionist perspective on knowledge, and based on “the assumption that a key feature of a claim’s epistemological status is its need to be certified by an appropriate social group before passing as knowledge”, as stated in the announcement of his new journal *Social Epistemology* in 1987. In this latter version, and as originally defined by Shera, social epistemology underpins the following four dimensions of our approach to the study of *evidence*, which are consistent with our focus on basic concepts as outlined above:

1. When exploring the concept of *evidence*, we are not only concerned with science but with the whole social body of knowledge.
2. We consider knowledge not merely as an individual but primarily as a social endeavour involving the whole social fabric.
3. We consider the epistemological questions of what can be known and how knowledge claims can be assessed as relative to the social processes in which the knowledge is produced. This is not the same as arguing that all knowledge is equally true; our argument, rather, is that truth is only one of the situated principles according to which knowledge claims are or can be assessed.
4. We approach the study of evidence from an *empirical* point of view by analysing and assessing how the concept is actually used in various discourses. As such, our approach relates to recent developments in the field of experimental medical philosophy, which share the overall ambition of promoting empirical methods within philosophy and the assumption that conceptual analysis can be informed by empirical data (Veit 2020). However, while our approach is empirical it is not bound to any experimental method, and where experimental philosophy has mostly used surveys, our study rather draws on corpus linguistics and conceptual history.

Combining this social epistemological perspective with corpus linguistics and elements of conceptual history, we argue that the concept of evidence should not be studied solely in the restricted environments that lay claim to a specific notion of it. Evidence can only be understood and assessed within the diverse discursive environments in which the concept actually operates.

**Fig. 1:**
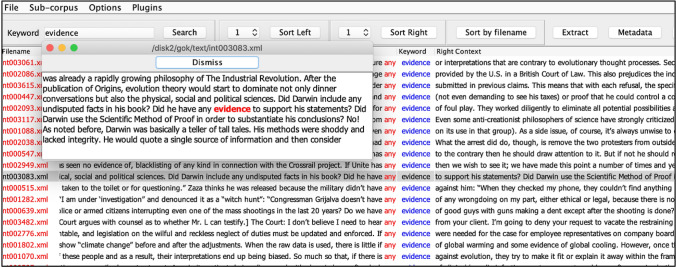
Example screenshot of a KWIC concordance of *evidence* from the GoK interface, ordered by the lexical item in position 1 to the left of the keyword

The data on which the analysis offered in “Analysis” section is based consists of the full Genealogies of Knowledge (GoK) corpus of Internet English. GoK is a multidisciplinary research project that has built corpora of ancient Greek, Latin, medieval Arabic and modern English. Each of these languages has served, at a particular point in time, as a *lingua franca* governing the intercultural circulation of knowledge. In the GoK browser environment, these corpora are queried separately, but their content is connected via translation and other forms of mediation, and thematically the corpora are all constructed around broad notions of scientific and political discourse. In other words, all texts contained in the corpora have contributed to our contemporary conceptual apparatus for expressing notions such as *expertise, proof*, and *evidence*, but also *justice, rights*, or *democracy*. For the English language, the corpus is divided into two main subcorpora. One corpus represents canonical academic knowledge, usually published in book form, whereas the other corpus represents a wide variety of publications gathered from the Internet, more specifically from media seeking to give voice to multiple opinions that contest the political and scientific consensus arguably found in traditional print and broadcast media. The texts in the GoK Internet corpus consist mostly of short articles and blog posts written from a polemic or activist perspective, published within the last 15 years. This corpus (totalling 5.6 million tokens) provides all data discussed below and includes a total of 3476 texts from over 35 outlets, including *ScienceBlogs*, *Discover Society*, *openDemocracy*, and *UCSUSA* (Union of Concerned Scientists).^3^ All texts are written in the English language, and the corpus is not meant to provide a representative overview of ‘the Internet’—something that would be impossible to attain. Rather, it provides a broad sample of Anglophone voices represented in online alternative media. The corpora compiled in the context of the GoK project are queried via a freely accessible concordance browser.^4^ The main interface is a classic keyword-in-context (KWIC) display, which returns concordance lines in response to a search for a given keyword; longer stretches of text are viewed by means of an ‘extract’ function, designed in compliance with copyright regulations (Fig. 1). In addition to the concordance interface, the software also offers a range of visualization tools (Luz and Sheehan 2020), some of which feature in the analysis we present here.

## Analysis

In assessing the controversy around the adequacy of EBM, Martini (2020) asserts that disagreement partly results from “ambiguity about the concept of evidence”, which often goes largely “unanalysed” and therefore “contains the contradictions that allow both camps to defend their positions and charge their adversaries”. Similar concerns about the lack of critical reflection on the meanings of *evidence* and *evidence-based* have been raised in relation to other areas of evidence-based practice, where analogous controversies have arisen. For instance, Kvernbekk (2011, p. 515) notes that *evidence-based* has become “a buzzword in contemporary educational debates (and also in medicine and policymaking, among other areas)”, and resorts to philosophy to unpack the meanings of the two components of the term, namely *evidence* and *based*. Rather than pursue this discussion in the realm of philosophical debate, our proposed methodology involves analysing attested uses of the two concepts that feed into different interpretations of *evidence-based medicine*—*evidence* and *based*—in a large electronic corpus of texts written by non-specialists using *evidence, -based,* and *evidence-based* in arguments about everyday topics such as climate change and human rights, as well as specialists on various areas of Evidence-based Practice, including EBM. Understanding the variety of meanings that basic concepts such as *evidence* assume in general discourse and in different contexts may afford us some insight into how they came to be condensed and streamlined to support a specific conception of medical practice associated with the EBM model and dominant among some, though by no means all members of the medical community.

### *Evidence* in the GoK Internet corpus

**Fig. 2:**
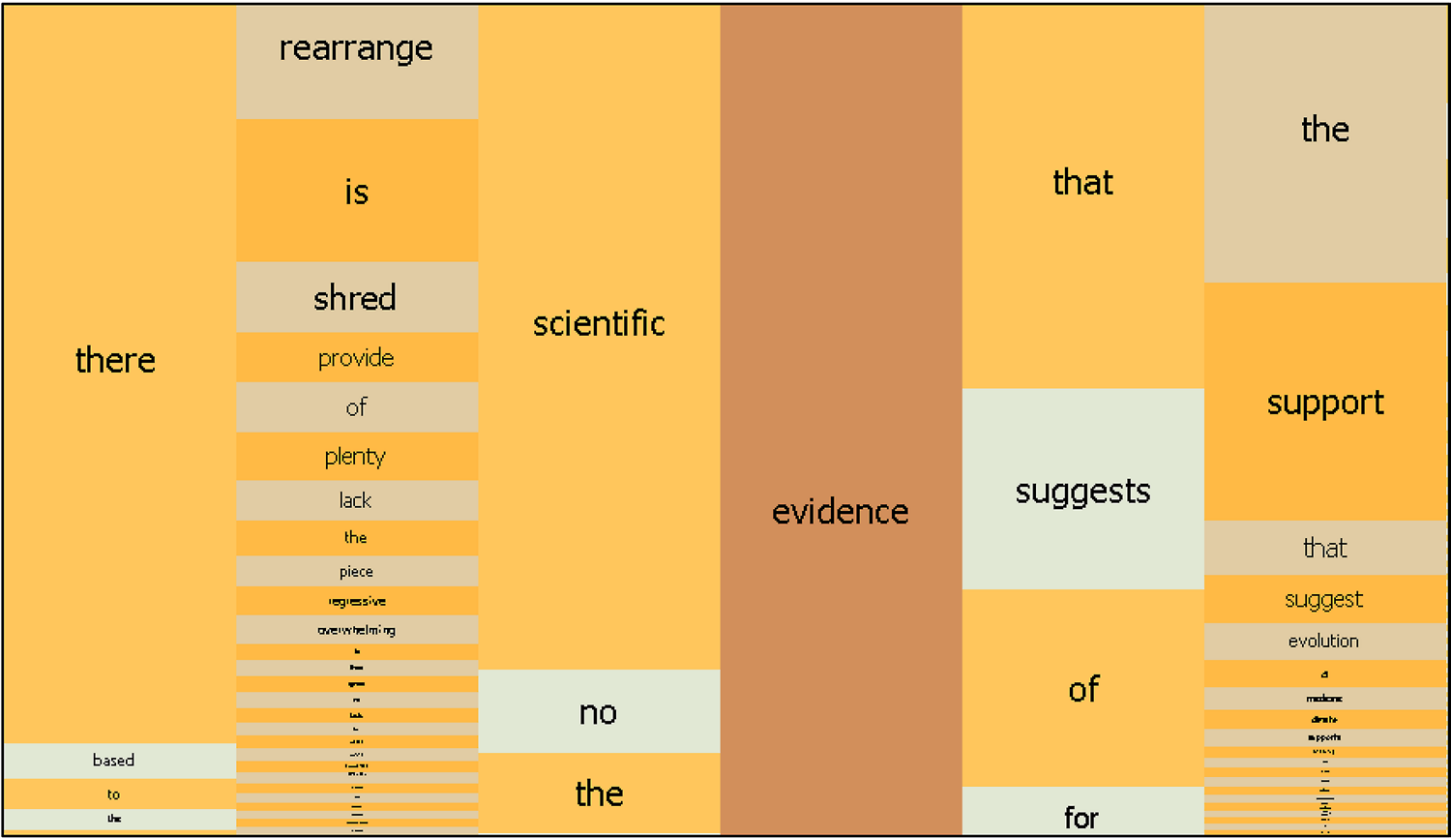
Mosaic of *evidence* collocates in the GoK Internet Corpus (cropped). The size of the tiles is relative to the words’ collocation strength, calculated using the MI3 measure (a variant of Mutual Information less biased towards rare words), omitting log transformation. Local view is selected within the interface, meaning that significance is represented within each position to the left or right of the keyword, rather than across all positions (global view). See Luz and Sheehan (2020, pp. 11–12) for a broader discussion of the visualization tool

In the GoK Internet corpus, there are 4402 occurrences of the keyword *evidence*, placing this basic concept in a very high (118th) position in the general frequency list. *Evidence* occurs with higher frequency in the corpus than lexical items that are very common throughout the English language, such as *same* and *since*, and is also more frequent than many other terms that encode basic concepts characteristic of our thematically designed corpus, such as *policy* and *society.* At N − 1, the position one word to the left of the keyword, the most frequent collocates are *the* (719), *of* (359) and *no* (229). *The* and *of* are the two most frequent words in the overall corpus, so their high frequency in relation to *evidence* is not immediately informative. In contrast, *no* generally occurs much less frequently, and thus the pair *no evidence* makes for an interesting collocation, as can be seen in Fig. 2.

Many instances of the collocation *no evidence* form part of the larger pattern *no evidence of* (64), a phrase that in turn occurs in several longer sequences, as in the following examples:

**There is no evidence of a link between** autisms and vaccines. (Hoofnagle and Hoofnagle 2008; Denialism Blog)

**There is no evidence of a link between** glyphosate in the food chain and autism, diabetes and obesity. (Zaruk 2016; The Risk Monger)

Both examples are taken from individual blogs concerned with a broad range of scientific matters, yet remarkably, the same exact phrasing occurs twice 8 years apart, and both times in an argument against purported connections between chemicals and health disorders. The first example speaks of a supposed link between autism and vaccination, and while no relevant causality has been established in the medical literature, belief in this relation seems to have only grown over the past decade and developed among large parts of the global population into a general aversion to vaccination. Indeed, while there are currently several vaccines available for COVID-19, this does not necessarily mean that the problem of ‘vaccine hesitancy’ is easily overcome (Harrison and Wu 2020). A central problem in this regard is that it may be feasible in the short term to provide evidence for the efficacy of a vaccine, but it is much harder to establish its safety. Indeed, variants of the common expression ‘absence of evidence is not evidence of absence’ occur several times in the corpus. The expression illustrates a logic on which the sceptic can always fall back when defending a suspicious position in relation to the long-term health effects of vaccines, or to any other hypothetical consequence of multi-faceted bodily or social interventions.

The second example intervenes in the glyphosate controversy, the debate about whether the use of this herbicide is harmful to humans. Glyphosate is domestically known for being the main active ingredient in Roundup, but is also used on a vast scale in agriculture. Studies on its effects are so far inconclusive. Chemical interactions produce complex effects, and while studies declaring the herbicide safe are often considered questionable for being produced by commercial stakeholders, several studies condemning the use of glyphosate are criticized for being “unsupported by evidence” (Mesnage and Antoniou 2017, p. 4). In this respect, providing evidence would mean incontestably showing that glyphosate in the environment causes disease. However, proponents of a stricter regulation of pesticide and herbicide use regularly invoke the “precautionary principle”: when in a state of “scientific uncertainty”, do not take risks (Kudsk and Mathiassen 2020, p. 216). The argument cannot easily be resolved, as both sides come to rely on the rhetorical fallacy termed *argumentum ad ignorantiam* (Walton 1999). Those critical of glyphosate argue that, since there is no proof of safety, the chemical is unsafe, whereas their opponents, in the absence of proven harmful effects, declare the herbicide safe. Thus, the burden of proof is passed back and forth, and a consensus cannot be reached. Consequently, further oppositional entrenchment can be expected, and notably, both articles from which the above examples are derived ultimately resort to name-calling: Hoofnagle and Hoofnagle (2008) frame the discourse of their opponents as “denialist claptrap”, and Zaruk (2016) seeks to confront what he calls “chemophobic propaganda”. Thus, the dichotomy established by the common pattern *no evidence* is indicative of a potential deadlock in scientific argumentation. If the idea of evidence requires absolute verifiability, its scope shrinks considerably. The fact that *the* collocates with *evidence* is also informative in this regard. The definite article, in contrast to the zero marker, implies certainty and singularity. *Evidence suggests* is not as forceful and monolithic as *the evidence suggests*, a phrase the high frequency of which signals, like *no evidence*, that the concept of *evidence* is often invoked when dealing in absolutes. One may also note, in this respect, a purported equivalence between *science* and *evidence* in parallel expressions of absolute certainty, as in the corpus examples *the science is clear* (7 instances) and *the evidence is clear* (9).

**Fig. 3:**
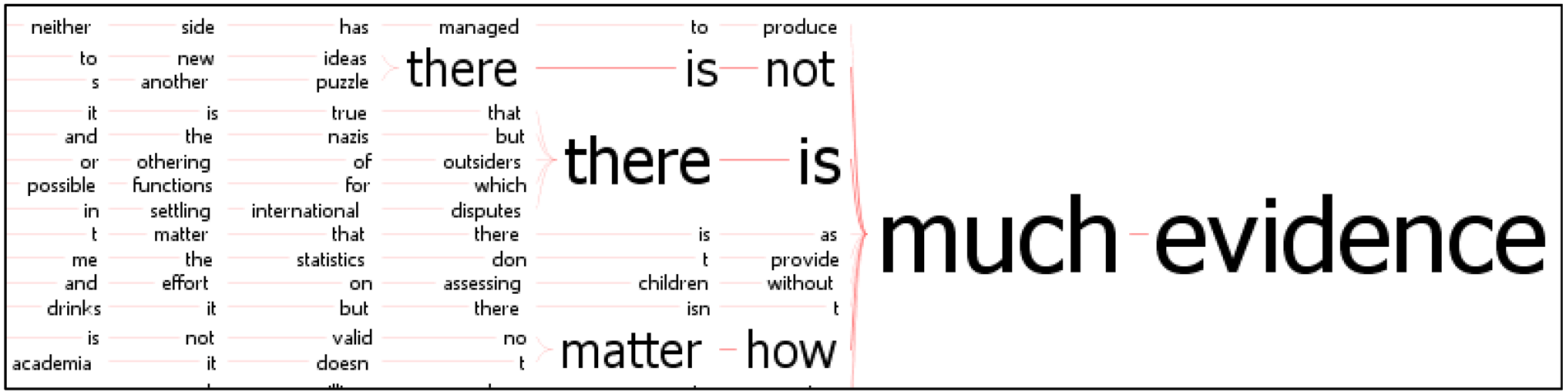
Concordance tree showing patterns preceding *much evidence* in the GoK Internet Corpus (cropped). Left co-text is shown. More common patterns are displayed in larger font. They include direct negation of sufficient quantity via the adverb not; more extended examples include ‘don’t provide’ and ‘neither side has managed to produce’. See Luz and Sheehan (2020, pp. 11, 13) for more information on the visualization tool

Other common collocates of *evidence* sketch a more nuanced picture, not restricted to the yes–no or present–absent binary: there can be *much* (17 instances) or *enough* (17) *evidence*, but also *insufficient* (17) or *little* (38) of it. The fact that the quantifiers expressing sufficiency are often negated, as in *not much evidence* or *not enough evidence*, while the main negative ones tend not to be reversed, suggests that the quantification of evidence in most cases implies that it falls short of confirming or supporting a given claim (Fig. 3).

Not only can evidence be quantified, but it can also be qualified in a variety of ways: *fossil evidence* (22) identifies a particular object of study, whereas *empirical evidence* (38) identifies a certain method of observation. *New evidence* (51) is uncovered in relation to earlier information on the same topic, and *supporting evidence* (11) is expected to back up claims to truth. An interesting category of qualifying adjectives concerns the specification of an affective response, as in *compelling* (29) and *convincing evidence* (13). *Convincing* is often modified by negation, as in *no convincing evidence*, whereas *compelling* and other intensifying qualifiers such as *ample* (14) and *damning* (7) are usually employed affirmatively, as in:

There is **compelling evidence** that children’s perceptions of cigarettes are influenced by branding and that branding detracts from the impact of health warnings on packs. (Abbott 2013; Left Foot Forward)

Note that the indefinite ‘there is’ does not specify where the evidence originates. While such specification may occur elsewhere in the article, a more impactful omission is that of the supposedly compelled subject—in the absence of a subject, the qualification of the *evidence* as *compelling* is presented as an objective characteristic, thus fabricating a tacit agreement between the author, their sources, and the implied reader. The implication is that the evidence ‘speaks for itself’. The representation of actors and participants is a central issue in critical discourse analysis (e.g. van Leeuwen 2008, pp. 23–54). In scientific as well as political reports, the responsibility of human agents is often elided, while “agency is shifted to abstract processes and entities” (Fairclough 2003, p. 138). In this regard, it is also important to take stock of the verbs of persuasion commonly found at the N + 1 position of *evidence*: the *evidence shows* (40), *indicates* (15) and *supports* (11). Together, these two patterns (the absence of a subject and the proliferation of verbs of persuasion in position N + 1) suggest that perspectives and positions are seemingly automatically validated by the presence of evidence, with no need for a human mediator to be specified as the agent responsible for interpreting the information at hand. The most common collocate on the right-hand side of *evidence*, namely *suggests* (59), forms part of this widespread grammatical pattern. Often, the broader pattern includes strong markers of emphasis that reinstate the yes–no dichotomy at the far ends of quantification: there are several variants of *all the evidence suggests* as well as *no evidence suggests*. In these cases, too, the majority of instances do not specify potentially relevant participants in the situation of evidence assessment.^5^

Rhetorically charged quantifications and qualifications such as *abundant* and *compelling* frequently precede *evidence*, but by far the most common qualifier modifying *evidence* is the seemingly more neutral adjective *scientific* (197). What exactly makes evidence scientific is seldom specified, but in some cases a number of expected characteristics are explicated:

This is the first **scientific evidence**, published in Environmental Research Letters, that confirms the numerous anecdotal accounts. (Albert et al. 2016; Desmog)

Here, scientific evidence is partly defined by the communicative setting: an academic outlet is required to present it, and it is contrasted to anecdotal accounts. The latter contrast implies, once again, demands of quantity and quality, as *anecdotal evidence* (14 instances) is generally understood to be gathered unsystematically and in limited amounts. Furthermore, anecdotes are told rather than observed, meaning that the scientific mode is presented in opposition to the narrative mode of exposition. While such schemas can in some cases be derived from the concordance lines, often the collocation *scientific evidence* is simply used as a means of emphasis, as further illustrated by the fact that the collocation is frequently preceded by additional adjectives of persuasion. Indeed, the most frequent pattern associated with *scientific evidence* is *overwhelming scientific evidence* (10 occurrences). *Overwhelming* encodes a rhetorical mixture of quantity and suggested affective response, and thus a merger of the features of adjectives such as *much* and *compelling*, discussed earlier. In addition, the phrase *overwhelming scientific evidence* is in the majority of cases used to set up a contrast, as in:

Despite the **overwhelming scientific evidence** linking fine soot particles to premature death, Honeycutt testified before Congress that “some studies even suggest PM makes you live longer.” (Negin 2017; UCSUSA)

Collocational patterns do not merely report a state of affairs; they express a communicative purpose which is of pragmatic rather than semantic importance (Stubbs 2009, pp. 124–125). In other words, recurrent phrases are likely to have an attitudinal or evaluative function that extends beyond their constative value. This function is termed semantic prosody in corpus linguistics, and in the case of the contrastive pattern *despite the overwhelming scientific evidence*, which occurs three times in the corpus, the function is not primarily to qualify the evidence in question but to suggest that one should be surprised at the existence of an opinion opposed to the author’s. Arguably, the various patterns of linguistic modification characteristic of *evidence* do not merely, or even primarily, serve to affirm, negate, qualify or quantify, but mainly to convince the implied reader of a given stance by rhetorical measures that conflate persuasive and epistemic modes of expression.

Importantly for our overall argument in this paper, when the GoK corpus is narrowed down to include only texts that make explicit mention of *evidence-based medicine*, the overall collocational patterns associated with *evidence* remain largely unchanged. Thus, the use of rhetorical adjectives such as *compelling* and *overwhelming* is not restricted to discussions outside the disciplinary scope of EBM, and this broad rhetorical inflection indicates that common discursive patterns of persuasion are shared across varied settings of practice. The boundaries of different spheres of knowledge production are porous, and basic concepts that display highly frequent patterns of usage alongside more specialist applications are particularly likely to generate heated disagreements through mutual misunderstanding. In the following section, we show how the meaning of *-based* is as elusive as that of *evidence*, further contributing to the fact that, while the concept of *evidence-based medicine* may purportedly apply to a restricted set of principles operational within a circumscribed context, this does not mean that it is necessarily presented and understood as such by different interlocutors.

### *Based* in the GoK Internet corpus

One of the central arguments put forward by corpus linguists is that meaning does not reside in individual words, but is realised in extended lexical elements. This means, as already shown in the previous section, that *evidence* has to be examined in its context of use. It also means that seemingly secondary elements, such as *based*, are bound to contribute heavily to the reception and interpretation of the concept and practice of *evidence-based medicine*. A search for *based* in the GoK Internet corpus returns 2891 concordance lines. *Based* is a multi-faceted lexical element that can fulfil a number of distinct linguistic functions. In order to focus on usages that structurally correspond to the phrase *evidence-based medicine*, we limit our analysis to its occurrence as part of a hyphenated compound adjective (as in *evidence-based*), rather than as a verb (as in *based on evidence*). However, the two variants are often closely related, and where relevant attested verbal uses will contribute to the argument.

For compound adjectives containing *based*, the Oxford English Dictionary (OED 2020) lists four main uses. A first common use indicates a relation to a specific location, as in *London-based* (36 instances) or *UK-based* (33). As these examples illustrate, such locations tend to be nations or cities. A second general use indicates a relation to a particular material, often described in chemical terms, and exemplified in the corpus by compounds such as *carbon*-*based* (10) and *chlorine-based* (1). The OED (2020) lists as a third option the use of *based* to indicate “a foundation, fundamental principle, or underlying basis”. This is by far the most frequent use in the corpus, not only because of the high frequency of *evidence-based* (218) and *science-based* (95), but also because of the widespread use of *based* in relation to social categorizations, as in *class-based* (36), *community*-*based* (30), or *faith-based* (25). The final main use listed by the OED concerns a specification of the base in question, as in *broad-based* (20). The fact that instances of all main categories of use can be easily retrieved from the concordance output suggests that the corpus provides an adequate general overview of the variety of meanings expressed by *based.*

The meaning of *based* cannot always be intuitively grasped by restricting one’s view to the various compounds in which it occurs, given that these compounds themselves form an adjectival unit that typically modifies a noun that follows. The co-text to the right of the compound has to be taken into account to arrive at an interpretation. A first observation is that when the adjectival compound itself contains a concrete material substance, the full phrase may still establish an abstract relation, as in *oil-based economy* (1) or *carbon-based capitalism* (1). The meaning of *based* is not equivalent in *carbon-based capitalism* and *carbon-based vegetation* (1), with the former expressing a relation of dependency and the latter one of composition. Thus, the interpretation of each adjective remains highly context dependent. An observation related to this tendency towards the abstract commonly expressed by *based* is the adjective’s frequent involvement in the description of large, intangible systems, which may be more or less regulated. The words *system* and *systems* immediately follow *based* fifteen times, and more specific examples of systems attested in this position include *taxation* (7), *politics* (8) and *organi[zs]ation(s)* (17). Social systems are composed of regular patterns of practice, and there is a marked tendency in the corpus for adjectival compounds containing *based* to be followed by nominal compounds containing *making*, such as *rulemaking* (follows *based* 7 times), *policymaking* (19) and *decision-making* (16, counts include hyphenated and unhyphenated spelling variants). This is a strong collocational pattern, as the gerundial form *making* seldom occurs in the corpus without the preceding words *policy* or *decision.* In fact, the expression *evidence-based decision-making* has become so cliché that, as the following example from our corpus indicates, it can be toyed with for humorous purposes:

Tom Slater has written that in order to give ‘scientific’ credibility to extending the neo-liberal state and deepening social inequality, think-tanks such as the CSJ have “mastered the craft of **decision-based evidence making**, tailored to the needs of policy elites and politicians on the lookout for accessible catchphrases to woo a jaded electorate.” (Silver et al. 2014; Discover Society)

Typically, nouns referring to the outcome of regulatory procedures such as *policymaking* can also follow *based*; there are, for instance, no fewer than 55 occurrences of *policy* or *policies* immediately following *based*, the bulk of which are part of the larger pattern *evidence-based policy* and *science-based policy*. Alternatives to *policy* that occur within similar patterns include *approach*(*es*) (21) and *practice*(s) (8). Thus, *based* very frequently, and often quite explicitly, functions to express the relation between a productive field of practice and the principles that guide it. This interpretation also holds for *evidence-based medicine,* yet what linguistically separates this particular phrase from its more general variants is the frequent use of an acronym, namely EBM (38 occurrences across several articles). There is, for instance, no acronym EBA, which could hypothetically refer to *evidence-based approaches*. The acronym EBP occurs only 17 times in the corpus, and its use is restricted to a single article (in which the P stands for *policy*, while outside the corpus the same abbreviation will often refer to *practice*). The use of acronyms is extremely common in scientific writing across various disciplines, even though in the health sciences it has been criticised as a source of “irritation, misunderstanding, and even alienation” (Moris 2020, p. 1274). Even writers who are seemingly well-acquainted with the vocabulary involved may produce formulations that suggest a lack of transparency, as the following example from the corpus illustrates:

One example is the implementation of a new procedure to treat asthma at the Royal Children’s Hospital in Victoria, Australia. The **EBM-based approach** was able to achieve an unusually high success rate of 95.5 percent during the first three months of transition. (Mawby and Harris 2016; OpenDemocracy)

In the above example, the abbreviation EBM itself contains the word *based*. Consequently, the example contains both a pleonastic and a tautological element, with the full formula *EBM* taking up the position normally reserved for *evidence*. The entire framework comes to serve as a substitute for the raw material: in other words, the wording may suggest that *EBM* occupies a singular, unambiguous conceptual space. This recalls our discussion of phrasings such as *the evidence suggests* in the previous section. *The evidence suggests* implies access to a fully transparent, univocal dataset, while *the EBM-based approach* implies access to a fully coherent, set framework, but in both cases the assumption of a rigid outline may be evaluated either positively or negatively. The author of the example above favours *EBM* and sees it as a model practice that should guide developments in other sectors. At the other end of the spectrum, some critics of the model characterise EBM’s rigid univocity as distinctly malignant, as the following example illustrates:

“[**EBM is not] medicine based on evidence**, but the equivalent in the field of medicine of a cult with its unique dogma, high priest … and fervent disciples,” says Dr. John Service, editor-in-chief of Endocrine Practice. (Hoofnagle 2007; Denialism)

The critique presented in this quote characterises *EBM* as an oppressive and disciplinary apparatus. Similar critiques were raised in other publications, including scholarly journals, around the same time the *Denialism* blog post was written. Holmes et al. (2006), for instance, argue that a focus on EBM “eliminates some ways of knowing”, a process which they ultimately describe as “microfascism”. This article too is quoted several times in the corpus. Importantly, in the quote above the speaker feels the need to unpack what he sees as the linguistic dishonesty of *EBM* by questioning the link to *evidence* as well as *based:* “[EBM is not] medicine based on evidence”*.* In effect he is suggesting that the verbal and the adjectival form of *based* should essentially express the same relation. Yet, in our corpus, the phrase *based on evidence* is largely reserved for unique events, decisions or explanations, and only seldom employed with the broad disciplinary quality of the related adjectival compounds, such as *evidence-based*. Disagreement on the proper application of terms such as *based* is thus at the heart of the argument. The quotes from both scholarly publications and more informal statements by specialists confirm that *evidence-based medicine* has met with strong resistance for over a decade now, while at the same time it is still presented as a model practice for other sectors to emulate. What some see as helpful instructions to guarantee best practice, others interpret as undue restrictions, and importantly, *based* elicits both meanings across the corpus, not just in discussions about the health sciences. Consider the following examples:

His book left scope for alternative views of meritocracy including positive associations of **merit-based** systems with social justice. (Gallinat 2018; Discover Society)

The corruption of visa work programs might soon expand under Trump’s immigration-reform agenda: His administration seeks to expand **“merit based**” migration of “high skill” and professional workers, while cutting humanitarian and family-reunification programs. (Chen 2017; The Nation)

In the first example, *based* connects a practice to its justification, while in the second it sceptically connects a practice to its limitations. The phrases are formally equivalent while their evaluative purposes—partially signalled through the use of scare quotes—are functionally opposed, as they are in different interpretations of *evidence-based medicine.* Thus, researchers seeking to untangle controversies in the health sciences may benefit from examining discursive patterns that cut across a broad range of disciplines and fields of practice. The continued rejection of the term *evidence-based* in the context of healthcare has of course not gone unnoticed, and in recent years alternative terms have come to circulate widely. The World Health Organization (n.d.), for instance, explicitly states that *evidence-based* implies a restricted viewpoint, and that the institution now prefers the term *evidence-informed*. It is not only *based* that in some contexts has fallen into disrepute. In Norwegian, the term *evidence-based* is often translated using *kunnskap*, usually an equivalent of *knowledge* rather than *evidence*, in part precisely to dissociate the discourse from the “positivist connotations that are tied to the English expression” (Bondevik and Engebretsen 2018). Yet, in all these examples, it is not so much the terms *evidence* or *based* that independently fall out of favour, but rather their specific combination, as the following example poignantly illustrates in the choice of different terms in parallel positions:

Ending AIDS requires **evidence-informed, rights-based** global leadership (Fried 2016; OpenDemocracy)

Yet are shifts in wording, such as the one from *evidence-based* to *evidence-informed*, indicative of a correspondent shift in practice? Positions on the subject will vary, but our corpus suggests that while *medicine* may attract a variety of modifiers such as a*lternative* (27 occurrences before *medicine*), *conventional* (8), *modern* (8) and *real* (8), none offer rival paradigms to *evidence-based*. They may refer to a competing outlook, but not to a competing set of principles and correspondent practices. The rigid view associated with *EBM* thus partly consolidates its continued dominance, at least as the standard against which to argue.

## Discussion

We began this article with a plea for scholars of the medical humanities to engage with a new research methodology we believe can shed light on important aspects of the evolution and contestation of a core constellation of concepts that underpin the practice and ethos of modern medicine. While the basic methodology of corpus-based CDA is well established in various areas of the social and human sciences, and to a much lesser extent in medicine, we set out to demonstrate that combining this methodology with theoretical assumptions drawn from conceptual history and social epistemology provides a powerful framework for elaborating an ambitious research agenda. We acknowledge the largely non-expert and diverse nature of our corpus and the need to supplement it with texts drawn specifically from the field of medicine. Our analysis is offered as a preliminary, pilot study that prepares the ground for the creation and analysis of large thematic corpora of expert and non-expert discourses on medicine, ranging from scientific papers published in journals such as *BMJ* and *The Lancet* to official WHO and CDC reports, medical textbooks, as well as blog posts and Wikipedia articles on medical topics. We hope to begin compiling these corpora in 2021, with a view to making them and an accompanying software interface available to the research community as soon as practicable. Creating such new resources can afford us novel insights into some of the continued controversies and points of contention in the field.

Focusing on one such point of contention, the controversy surrounding different interpretations of EBM, we argued, firstly, that it is in the nature of *evidence* as a basic concept with wide currency in both specialist and general language to support a variety of often conflicting meanings, and therefore to continue to invite contestation. We supported our argument with an analysis of the lexical patterning of *evidence*, followed by an analysis of *based,* the second component of EBM and a modifier that may activate a range of different, and conflicting associations. Drawing on a large corpus of Internet English featuring articles and blogposts written by medical practitioners, scientists, journalists and activists of various political hues, we unpacked some of the many meanings associated with each lexical item, highlighting the futility of attempting to impose a single, rigid interpretation of what *evidence-based* means in the context of modern medicine. The lexical patterns we identified had implications beyond their use in a particular blogpost or by a particular medical specialist or layperson. For example, one of the patterns we identified across texts and authors is *there is no evidence of*. The same pattern was documented decades ago in an early corpus-based study as prevalent in medical case reports, more specifically in the context of “eliminating unsubstantiated interpretations of a symptom or disease in order to narrow down the potential number of causes” (Baker 1988, p. 103). In the context of medical journal articles, the phrase concluded a specific investigation, the steps of which could be systematically traced back and verified. By contrast, when used in a polemical blogpost, the boundaries of the domain covered by a declaration that evidence is absent are not always clear, and instead of a formulaic statement of academic expression, the phrase becomes pre-emptive and axiomatic. This pattern may convey an epistemic sense of certainty, despite no verifiable conditions being in place to assess its situated truth value. In other words, a speaker’s purpose determines the patterns used, but patterns may be exchanged across different communicative settings, and thus fulfil different functions in specific contexts, while nevertheless retaining the semblance of a shared argumentative value across all those contexts.

The point is not to suggest that scholarly articles are fundamentally more transparent than blogposts in their use of *evidence*, but to elucidate why heated discussion may arise around ways of speaking about evidence and other basic concepts: when the same means of expression are used to discuss different modes of practice, interlocutors’ epistemic expectations may be violated. As both specialists and non-specialists move back and forth across different domains and are exposed to different interpretations of a basic concept, it is possible that those who reject whole paradigms of knowledge such as EBM may do so because they see them as presenting either too rigid or an overly flexible interpretation of the concept in question in relation to their own experience of its active use in a variety of contexts. Academics and professionals concerned with the promotion or contestation of medical paradigms therefore cannot ignore existing notions of evidence that circulate among different groups in society.

Our analysis also supports some of the basic presuppositions underpinning the social epistemology approach, as outlined in our introduction. First, the lexical patterns we identified had implications beyond their use in particular communicative settings and across the entire social fabric. Considering the nature of *evidence* as a basic concept, we need to draw on a theoretical approach that incorporates both scientific *and* lay processes of conceptualizing and evaluating evidence. Second, examining concordance patterns means that individual knowledge becomes secondary to discursive manifestations of knowledge as a social, linguistically negotiated endeavour. Third, we have found that knowledge claims can rest, as in the case of *evidence*, on attributions of sufficient quality or quantity, but that such assessments are always subject to rhetorical techniques of persuasion. We have also shown that elements such as *based*, which may appear to be neutral or insignificant, in fact express a wide range of divergent relations. Kvernbekk (2011, pp. 522–524) has argued that the meaning of *based* may indeed be unclear across various notions of evidence-based practice. Our analysis has shown that conflicts of interpretation may arise between social settings and knowledge domains that alternatively understand the relation expressed in *evidence-based* as one of disciplinary ties, one of institutional embedding, one of fundamental principles, or one of shared practices and goals. This suggests that a robust theory of evidence-based medicine needs to account for the values and principles that are specific to the discourse within which claims and beliefs about evidence are articulated. As also pointed out by other authors (Engebretsen et al. 2016), the EBM conception of knowledge fails to acknowledge that the way different groups engage in the process of knowing—as unfolding in specific settings and articulated in different types of discourse—determines the principles and objects of their knowledge. There is a need for a situated epistemological approach to EBM that recognizes and explains different types of rationality, and hence plural conceptualizations of *evidence* incorporating various interpretations and nuances of meaning that circulate in the everyday linguistic environment. To develop such a theory will be one of the aims of our future research.

## Footnotes

1 In CDA and beyond, criticism of corpus-based research generally focuses on the disproportionate attention paid to single words and phrases rather than larger textual units, and on the difficulty of convincingly relating textual patterns to extra-textual realities. In addition to linguistic and cultural decontextualization, the proper use of quantification and visualisation techniques is heavily debated. Taylor and Marchi (2018) offer a constructive overview of abiding methodological issues.

2 It also goes beyond some of the corpus-based studies in the philosophy of science that focus on differences in writing styles between historical and experimental sciences (Argamon et al. 2005; Argamon and Dodick 2006).

3 The full contents the corpus can be consulted at http://genealogiesofknowledge.net/corpora/internet-corpus/. Corpus building is still ongoing, but upon request the authors can provide a list of all articles included at the time of writing.

4 http://genealogiesofknowledge.net/software/.

5 A notable exception to this overall tendency is the pattern of appropriation *our evidence suggests* (6 instances). This pattern partly intimates the revelatory quality of investigative journalism, but it also strongly suggests institutional ownership—an undifferentiated internet search for *our evidence* will mostly return information from large health-related research bodies such as *Cochrane* and *BMJ*.
